# High platelet reactivity affects the clinical outcomes of patients undergoing percutaneous coronary intervention

**DOI:** 10.1186/s12872-016-0394-0

**Published:** 2016-11-29

**Authors:** Jun-Jie Zhang, Xiao-Fei Gao, Zhen Ge, Nai-Liang Tian, Zhi-Zhong Liu, Song Lin, Fei Ye, Shao-Liang Chen

**Affiliations:** 1 Department of Cardiology, Nanjing First Hospital, Nanjing Medical University, No. 68 Changle road, 210006 Nanjing, China; 2 Department of Cardiology, Nanjing Heart Center, Nanjing, China

**Keywords:** Platelet function test, High platelet reactivity, Drug eluting stent, Stent thrombosis

## Abstract

**Background:**

The association of platelet reactivity and clinical outcomes, especially stent thrombosis, was not so clear. We sought to investigate whether high platelet reactivity affects clinical outcomes of patients with drug eluting stents (DESs) implantation.

**Methods:**

All enrolled individuals treated with DESs implantation were evaluated by PL-11, using sequentially platelet counting method. The primary end point was the occurrence of definite and probable stent thrombosis at 2 years. The secondary endpoint was major adverse cardiovascular and cerebrovascular events (MACCE), including all cause death, spontaneous myocardial infarction (MI), target vessel revascularization (TVR), and ischemic stroke.

**Results:**

A total of 1331consecutive patients were enrolled at our center. There were 91 patients (6.8 %) identified with high platelet reactivity (HPR) on aspirin, and 437 patients (32.9 %) with HPR on clopidogrel. At 2-year follow-up, the incidence of stent thrombosis was significantly higher in patients with HPR on aspirin (9.9 % vs. 0.4 %, *p* < 0.001), and HPR on clopidogrel (3.0 % vs. 0.1 %, *p* < 0.001). There were increased MACCE in the HPR on aspirin group (16.5 % vs. 8.5 %, *p* = 0.021), mainly driven by the higher all cause death (7.7 % vs. 1.6 %, *p* = 0.002) and MI (9.9 % vs. 1.9 %, *p* < 0.001) in the HPR on aspirin group. Similarly, the rate of MACCE was higher in the HPR on clopidogrel group (12.4 % vs. 7.4 %, *p* = 0.004). No differences in all bleeding and hemorrhagic stroke were observed.

**Conclusions:**

The present study demonstrated that high platelet reactivity on both aspirin and clopidogrel were associated with incremental stent thrombosis following DESs implantation.

**Electronic supplementary material:**

The online version of this article (doi:10.1186/s12872-016-0394-0) contains supplementary material, which is available to authorized users.

## Background

Stent thrombosis was recognized as an important complication of percutaneous coronary intervention (PCI), ranging around 0.5 to 2 % with the use of drug-eluting stents (DES) [1, 2], but stent thrombosis had a high risk of myocardial infarction and cardiac death [3, 4]. Effective dual anti-platelet therapy (DAPT) with aspirin and clopidogrel is mandatory after DES implantation, due to DES inducing platelet adhesion, activation and thrombus formation. Anti-platelet insufficiency, especially high platelet reactivity on aspirin and clopidogrel, has been considered to play an important role in stent thrombosis [5].

There are several studies exploring the association between platelet reactivity and stent thrombosis [5–8], however, some limitations still existed, such as low-risk population, low event rates, and conflicted results. Moreover, the VerifyNow P2Y12 assay was widely used in these studies, which has been reported to be nonflexible, very expensive, limited hematocrit and platelet count, and just moderate agreement with other platelet function tests [9, 10]. However, sequentially platelet counting method by PL-11 (SINNOWA Co., Nanjing, China) was a novel analyzer for platelet reactivity test by automatic impedance technique [11]. Therefore, this prospective study was designed to assess the association between platelet reactivity with the novel automatic platelet aggregometer and stent thrombosis among patients with DES implantation.

## Methods

### Study population

**Fig. 1:**
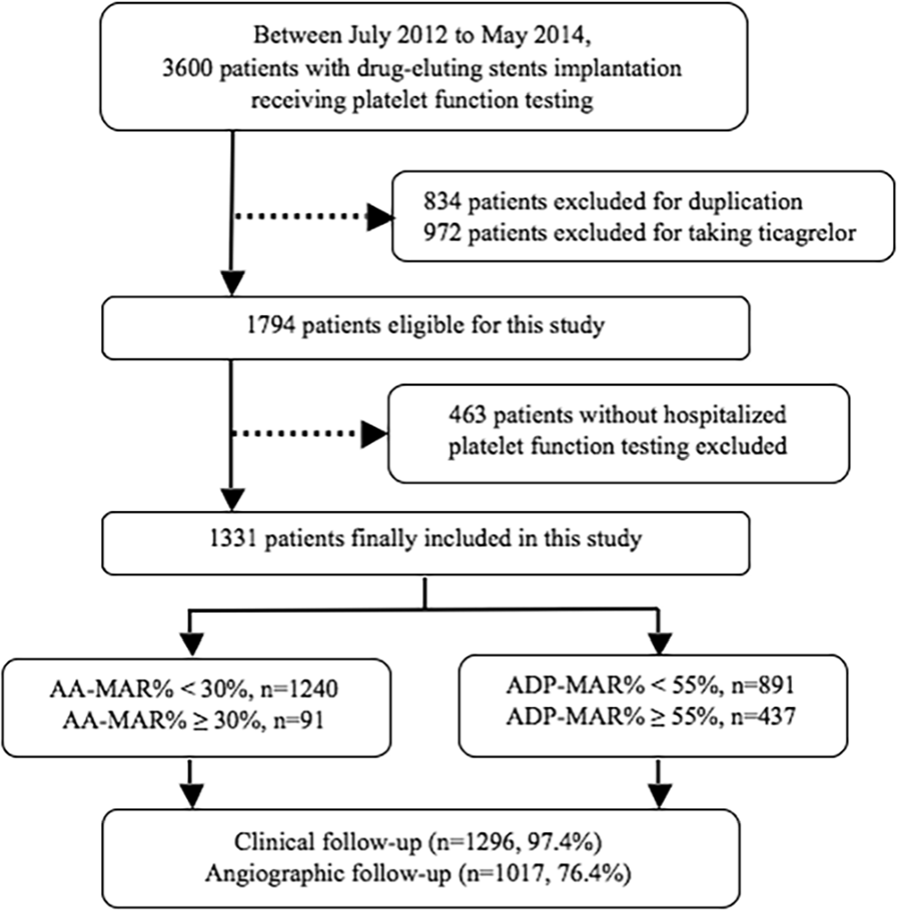
Flowchart of study design. The value of ADP-MAR% was missed in three patients. AA: arachidonic acid; MAR: maximal aggregation ratio; ADP: adenosine diphosphate

From July 2012 to May 2014, a total of 3600 consecutive real-world patients who treated with DES implantation from our center were considered candidates for this registry. Finally 1331 patients were included in this study according to the inclusion and exclusion criteria. Inclusion criteria were as follows: age >18 years, and successful PCI in at least one major epicardial coronary artery. Exclusion criteria were demonstrated in Fig. 1. Additional exclusion criteria included pregnancy, a platelet count < 10*10^9^ /L and suspected intolerance to one of the study drugs.

### Interventional procedure and medications

All interventional procedures were performed in accordance with the current guidelines. The type of DES selection, procedural technique, use of glycoprotein IIb/IIIa inhibitors, intravascular ultrasound, fractional flow reserve, and optical coherence tomography were at the discretion of the operators. A loading dose of aspirin and clopidogrel of at least 300 mg were administered prior to the index procedure. Heparin was used during the procedure to maintain an activated clotting time more than 250 s. Total creatine kinase (CK), CK-MB, and troponin were dynamically measured until 72 h post-procedure. After the intervention, all patients received 100 mg/day aspirin for life and clopidogrel (75 mg/day) for at least 12 months.

### Platelet function test

Platelet reactivity was assessed in all patients before heparin administered during procedure in the Lab room. PL-11 analyzer (SINNOWA Co., Nanjing, China) was used for the test [11, 12]. With standardized and easier test method, the analyzer gives more exact and stable results of platelet maximum aggregation ratio (MAR%). PL-11 analyzer measures the platelet function by “sequentially platelet counting method”, and the analyzer automatically and sequentially counts (impedance technology) the numbers of platelet in the citrated whole blood sample before and after adding agonists in fixed time interval during the whole testing progress. For each agonist test, 500 ul citrated whole blood sample was transferred into a test tube, then it was placed into the test position in the analyzer, press the “test button” the analyzer begins to count the platelet of the sample automatically two times and calculates the original platelet number (mean value) by the first two counts, then 40 ul of agonist was added into the sample and mixed to activate platelets aggregating. The numbers of platelet in the sample will be decreased because aggregation. More platelet aggregated, lower numbers of platelet existing in the sample. After adding agonist, the analyzer continues to measure platelet numbers in the sample for 3 times, also in fixed time interval and get three results of platelet number, the lowest platelet number of the three is used to calculate the MAR% in the following formula: (1-lowest platelet number/initial platelet number) × 100 %. The whole test progress is 12 min, the sample position keeps at 37 °C and keeps swaying during the progress, so the sample is kept in well mixed and good condition during testing. Arachidonic acid (AA, 2 mg/ml) and adenosine diphosphate (ADP, 50umol/L) were used as agonists. Additionally, high platelet reactivity (HPR) on aspirin was defined as MAR% ≥ 30 %; meanwhile, HPR on clopidogrel was defined as MAR% ≥ 55 %. All investigators were strongly encouraged not to change antiplatelet strategy according to the results of platelet function test.

### Study endpoints and definitions

The primary end point was the occurrence of definite and probable stent thrombosis at 2 years, defined according to the Academic Research Consortium (ARC) classification [13]. The secondary endpoint was the incidence of major adverse cardiovascular and cerebrovascular events (MACCE), including all cause death, spontaneous myocardial infarction (MI), target vessel revascularization (TVR), and ischemic stroke. The safety endpoint was the risk of all bleeding and hemorrhagic stroke, defined according to the Bleeding Academic Research Consortium (BARC) classification [14]. All deaths were considered cardiac in origin unless a non-cardiac cause was confirmed clinically or at autopsy. Spontaneous MI was diagnosed in accordance with Third Universal Definition of Myocardial Infarction [15]. Target lesion revascularization and TVR were defined as repeat revascularization (including PCI and coronary artery bypass grafting) for target lesions and target vessels, respectively, in the presence of symptoms or objective signs of ischemia. Stroke was defined as global or focal cerebral, spinal cord, or retinal injury resulting in acute neurological dysfunction and was further classified into ischemic and hemorrhagic.

### Follow-up

Clinical follow-up was performed either by telephone or through a clinical office visit at 1, 6, 12, and 24 months. Repeat coronary angiography was scheduled at 13 months after the indexed procedure unless clinical reasons indicated earlier. All clinical events were assessed by an independent committee that was blinded to the study.

### Statistical analysis

The distribution of continuous variables was assessed by the Kolmogrov-Smirnov test. Categorical variables were expressed as frequencies or percentages and compared by Chi-square statistics or Fisher’s exact test. Continuous variables were summarized as means ± standard deviation (SD) or median and compared using Students’ *t*-test (for normal data) and Mann–Whitney *U*-test (for non-normally distributed variables). Survival curves with time-to-event data were generated by the Kaplan-Meier method and compared using the log-rank test. Hazard ratios (HR) are presented along their 95 % confidence interval (CI). Multivariable Cox proportional hazard model including platelet reactivity, other clinical and procedural variables was applied to identify the independent predictors that correlated with definite and probable stent thrombosis. Receiver-operating characteristic (ROC) curves were generated to assess the association between platelet reactivity and clinical outcomes. A *p* value <0.05 was considered statistically significance. All analyses were performed with the use of the statistical program SPSS 22.0 (SPSS Institute Inc, Chicago, Illinois).

## Results

### Baseline clinical characteristics

A total of 1331 patients with DES implantation were finally enrolled, and baseline clinical characteristics were showed in Table 1. The median AA-MAR% of 1331 enrolled patients was 18.2 %, and the median ADP-MAR% of 1328 patients was 36.4 %. There were 91 patients (6.8 %) identified with HPR on aspirin, and 437 patients (32.9 %) with HPR on clopidogrel. 86.2 % of the individuals were admitted with acute coronary syndromes (ACS). Baseline clinical characteristics between HPR on aspirin group and normal platelet reactivity (NPR) on aspirin group were comparable. Patients in the NPR on clopidogrel group were more likely males (77.7 %) and ACS (87.8 %), when compared to the HPR on clopidogrel group (70.9 %, *p* = 0.008; 82.8 %, *p* = 0.018).

**Table 1:** Baseline clinical characteristics

	NPR on aspirin (*n* = 1240)	HPR on aspirin (*n* = 91)	*P* value	NPR on clopidogrel (*n* = 891)	HPR on clopidogrel (*n* = 437)	*P* value
Age, yrs	64.19 ± 10.06	65.63 ± 9.75	0.189	63.96 ± 10.23	64.98 ± 9.60	0.082
Male, *n* (%)	936 (75.5)	67 (73.6)	0.706	692 (77.7)	310 (70.9)	0.008
BMI, kg/mm^2^	24.70 ± 3.01	24.55 ± 3.01	0.649	24.65 ± 2.95	24.78 ± 3.12	0.449
Hypertension, *n* (%)	888 (71.6)	66 (72.5)	0.905	644 (72.3)	307 (70.3)	0.438
Hyperlipidemia, *n* (%)	318 (27.4)	23 (25.6)	0.806	236 (28.3)	105 (25.4)	0.281
Diabetes, *n* (%)	328 (26.5)	24 (26.4)	NS	239 (26.8)	112 (25.6)	0.691
Current smoking, *n* (%)	482 (39.2)	33 (36.7)	0.656	356 (40.3)	159 (36.7)	0.229
ACS, *n* (%)	1072 (86.5)	75 (82.4)	0.272	782 (87.8)	362 (82.8)	0.018
eGFR < 60 ml/min/1.73 m^2^, *n* (%)	122 (10.1)	8 (9.2)	NS	94 (10.9)	36 (8.4)	0.170
Prior stroke, *n* (%)	138 (11.2)	11 (12.2)	0.731	93 (10.5)	56 (12.9)	0.196
Prior PCI, *n* (%)	236 (19.2)	15 (16.7)	0.676	161 (18.2)	89 (20.6)	0.331
LVEF, %	59.80 ± 9.33	58.50 ± 9.89	0.275	59.59 ± 9.27	59.94 ± 9.61	0.587
hsCRP, ug/ml	31.94 ± 8.88	32.84 ± 9.16	0.400	31.98 ± 8.80	32.13 ± 9.11	0.804
Agents in hospital						
Aspirin, 100 mg/d	1216 (98.1)	90 (98.9)	NS	881 (98.9)	428 (97.9)	0.218
Clopidogrel, 75 mg/d	1203 (97.0)	88 (96.7)	0.751	863 (96.9)	426 (97.5)	0.606
Agents at 2 years						
Aspirin (100 mg/d), *n* (%)	1142 (92.1)	81 (89.0)	0.317	800 (89.8)	398 (91.1)	0.493
Clopidogrel (75 mg/d), *n* (%)	806 (65.0)	57 (62.6)	0.650	570 (64.0)	266 (61.9)	0.277

*BMI* body mass index, *ACS* acute coronary syndrome, *eGFR* estimated glomerular filtration rate, *PCI* percutaneous coronary intervention, *LVEF* left ventricular ejection fraction, *hsCRP* high sensitivity C reactive protein, *NPR* normal platelet reactivity, *HPR* high platelet reactivity

### Lesions and procedural characteristics

No differences in the lesions and procedural characteristics were observed between HPR on aspirin and NPR on aspirin group (Table 2). In addition, there were also no differences between HPR on clopidogrel and NPR on clopidogrel group, except for more frequent use of glycoprotein IIb/IIIa Inhibitors for patient with NPR on clopidogrel (12.0 % vs. 8.2 %, *p* = 0.048).

**Table 2:** Angiographic and procedural characteristics

	NPR on aspirin (*n* = 1240)	HPR on aspirin (*n* = 91)	*P* value	NPR on clopidogrel (*n* = 891)	HPR on clopidogrel (*n* = 437)	*P* value
Multi-vessel disease, *n* (%)	517 (59.2)	41 (63.1)	0.601	363 (59.6)	194 (59.1)	0.889
Bifurcation lesion, *n* (%)	407 (32.8)	35 (38.5)	0.300	301 (33.8)	141 (32.3)	0.578
Thrombus-containing lesions, *n* (%)	113 (9.3)	5 (5.7)	0.336	77 (8.9)	41 (9.5)	0.683
IIb/IIIa Inhibitor, *n* (%)	122 (10.8)	7 (8.6)	0.709	96 (12.0)	33 (8.2)	0.048
DES used, *n* (%)	1240 (100)	91 (100)	NS	891 (100)	437 (100)	NS
Total implanted stent	2.78 ± 1.72	2.58 ± 1.61	0.287	2.81 ± 1.76	2.69 ± 1.62	0.235
Mean stent diameter, mm	3.08 ± 0.38	3.03 ± 0.34	0.313	3.08 ± 0.38	3.05 ± 0.39	0.155
Total stent length, mm	72.14 ± 48.69	65.06 ± 42.73	0.187	72.61 ± 49.14	69.77 ± 46.65	0.332
Complete revascularization, *n* (%)	823 (67.9)	59 (67.0)	0.906	598 (68.8)	281 (65.7)	0.256
Final TIMI grade 3, *n* (%)	1199 (98.2)	88 (98.9)	NS	860 (98.3)	424 (98.1)	0.827
Heparin volume, units	7915.11 ± 2027.05	8085.00 ± 1945.01	0.503	7892.79 ± 2029.11	7999.97 ± 2012.82	0.421

*DES* drug-eluting stent, *TIMI* thrombolysis in myocardial infarction, *NPR* normal platelet reactivity, *HPR* high platelet reactivity

### High platelet reactivity and clinical outcomes

**Fig. 2:**
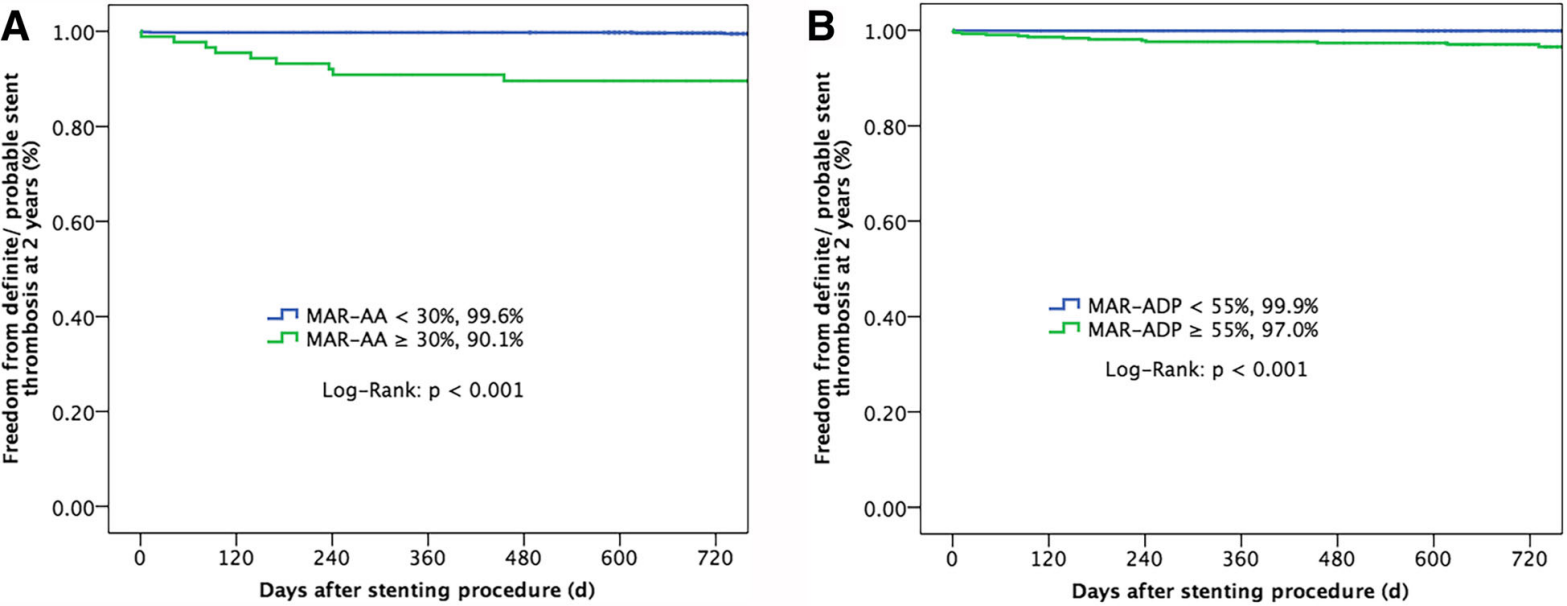
Definite/ probable stent thrombosis-free survival rate at 2 years. Freedom from definite/probable stent thrombosis at 2-year follow-up**a** between AA-MAR% ≥ 30 % (*green line*) and AA-MAR% < 30 % (*blue line*), **b** between ADP-MAR% ≥ 55 % (*green line*) and ADP-MAR% < 55 % (*blue line*). Abbreviations are showed in Fig. 1

After a median follow-up of 2 years, there were 9 (9.9 %) definite and probable stent thrombosis events in the HPR on aspirin group and 5 (0.4 %) in the NPR on aspirin group (*p* < 0.001, Table 3, Fig. 2a), with the detailed information of these 14 patients listed in Table 4. The risk of stent thrombosis in the HPR on clopidogrel group was 3.0 %, much higher than 0.1 % in the NPR on clopidogrel group (Fig. 2b). By Cox regression multivariable analysis, the independent predictors of definite and probable stent thrombosis were AA-MAR% (HR: 1.844, 95 % CI: 1.348 to 2.522, *p* < 0.001), ADP-MAR% (HR: 1.680, 95 % CI: 1.128 to 2.502, *p* = 0.011), and total implanted stents (HR: 1.421, 95 % CI: 1.108 to 1.822, *p* = 0.006).

**Table 3:** Clinical outcomes

	NPR on aspirin (*n* = 1240)	HPR on aspirin (*n* = 91)	*P* value	NPR on clopidogrel (*n* = 891)	HPR on clopidogrel (*n* = 437)	*P* value
In hospital						
Definite/probable Stent thrombosis	1	0	NS	0	1	0.329
All cause death	0	0	NS	0	0	NS
Cardiac death	0	0	NS	0	0	NS
Spontaneous MI	2	0	NS	0	2	0.108
TLR	1	0	NS	0	1	0.329
TVR	1	0	NS	0	1	0.329
Ischemic stroke	8 (0.6)	0	NS	4 (0.4)	4 (0.9)	0.451
All bleeding	39 (3.1)	3 (3.3)	0.762	30 (3.4)	12 (2.7)	0.619
Hemorrhagic stroke	3 (0.2)	0	NS	3 (0.3)	0	0.555
MACCE	10 (0.8)	0	NS	4 (0.5)	6 (1.4)	0.090
At 1 years						
Definite/probable Stent thrombosis	3 (0.2)	8 (8.8)	< 0.001	1 (0.1)	10 (2.3)	< 0.001
All cause death	8 (0.6)	2 (2.2)	0.146	3 (0.3)	7 (1.6)	0.018
Cardiac death	2 (0.2)	2 (2.2)	0.025	1 (0.1)	3 (0.7)	0.107
Spontaneous MI	17 (1.4)	3 (3.3)	0.152	10 (1.1)	10 (2.3)	0.147
TLR	30 (2.4)	0 (0)	0.260	25 (2.8)	5 (1.1)	0.075
TVR	31 (2.5)	0 (0)	0.265	25 (2.8)	6 (1.4)	0.123
Ischemic stroke	9 (0.7)	1 (1.1)	0.509	9 (1.0)	1 (0.2)	0.180
All bleeding	64 (5.2)	3 (3.3)	0.619	48 (5.4)	19 (4.3)	0.505
Hemorrhagic stroke	5 (0.4)	0	NS	3 (0.3)	2 (0.5)	0.666
MACCE	40 (3.2)	5 (5.5)	0.228	30 (3.4)	15 (3.4)	0.951
At 2 years						
Definite/probable Stent thrombosis	5 (0.4)	9 (9.9)	< 0.001	1 (0.1)	13 (3.0)	< 0.001
All cause death	20 (1.6)	7 (7.7)	0.002	3 (0.3)	23 (5.3)	< 0.001
Cardiac death	4 (0.3)	7 (7.7)	< 0.001	1 (0.1)	10 (2.3)	< 0.001
Spontaneous MI	23 (1.9)	9 (9.9)	< 0.001	11 (1.2)	21 (4.8)	< 0.001
TLR	60 (4.8)	5 (5.5)	0.799	51 (5.7)	14 (3.2)	0.057
TVR	65 (5.2)	5 (5.5)	0.809	51 (5.7)	19 (4.3)	0.360
Ischemic stroke	16 (1.3)	2 (2.2)	0.352	12 (1.3)	6 (1.4)	NS
All bleeding	118 (10.0)	8 (9.2)	NS	93 (10.9)	33 (7.9)	0.110
Hemorrhagic stroke	8 (0.6)	0 (0)	NS	4 (0.4)	4 (0.9)	0.451
MACCE	106 (8.5)	15 (16.5)	0.021	66 (7.4)	54 (12.4)	0.004

*MI* myocardial infarction, *TLR* target lesion revascularization, *TVR* target vessel revascularization, *MACCE* adverse cardiovascular and cerebrovascular events, *NPR* normal platelet reactivity, *HPR* high platelet reactivity

**Table 4:** Characteristics of patients with definite and probable stent thrombosis

Case	Risk factors	Lesions	Stent number	AA-MAR, %	ADP-MAR, %	CYP2C19 gene	Use of antiplatelet drugs	Time from PCI to stent thrombosis (days)	Cardiac death
1	EH	Multi-vessel disease	2	15.4	60.6	NA	Dual-antiplatelet for 2 years	2	0
2	Smoking	Left main lesion	3	42.9	60.3	NA	Dual-antiplatelet for 2 years	241	1
3	EH, DM, smoking	Multi-vessel disease	8	35	58.4	Poor metabolizer	Dual-antiplatelet for 2 years	170	0
4	EH, smoking	Multi-vessel disease	3	20	58.6	NA	Dual-antiplatelet for 2 years	1	0
5	DM	Multi-vessel disease	3	79.8	67.9	NA	Clopidogrel for lifelong time, aspirin for half year	236	1
6	EH, smoking	Pro-LAD lesion	1	50	60.5	Intermediate metabolizer	Clopidogrel for 1 year, aspirin stopped by oneself at 1 month after procedure, and aspirin re-used at 1 year	455	0
7	EH	Multi-vessel disease	3	20.2	63	Intermediate metabolizer	Aspirin for lifelong time, clopidogrel for 1 year	12	0
8	EH, DM	Multi-vessel disease	2	39	58.7	Intermediate metabolizer	Dual-antiplatelet for 2 years	82	0
9	No	Multi-vessel disease + left main lesion	3	49.8	61.1	NA	Dual-antiplatelet for 2 years	94	1
10	EH, DM	Multi-vessel disease + left main lesion	6	37	45	Poor metabolizer	Dual-antiplatelet for 2 years	1	0
11	DM, smoking	Multi-vessel disease	4	24.6	56.4	Poor metabolizer	Dual-antiplatelet for 2 years	731	1
12	EH, DM, smoking, CKD 4	Multi-vessel disease + left main lesion	7	21.1	68	Intermediate metabolizer	Dual-antiplatelet for 2 years	617	1
13	Smoking	Multi-vessel disease	2	34.6	57.2	Intermediate metabolizer	Dual-antiplatelet for 2 years	42	1
14	EH	Multi-vessel disease	6	45.4	60.3	Intermediate metabolizer	Dual-antiplatelet for 2 years	138	1

*EH* essential hypertension, *DM* diabetes mellitus, *CKD* chronic kidney disease, *LAD* left anterior descending, *AA* arachidonic acid, *MAR* maximal aggregation ratio, *ADP* adenosine diphosphate, *PCI* percutaneous coronary intervention

The area under ROC (AUC) of AA-MAR% predicting stent thrombosis was 0.808 (95 % CI: 0.695, 0.922; *p* < 0.001), with the cutoff value of 34.5 % (sensitivity: 64.3 %, specificity: 92.1 %, Additional file 1: Figure S1), which was comparable with the AUC of ADP-MAR% (0.747, 95 %: 0.623, 0.870, *p* = 0.001; *p* = 0.087 for AUC comparison), with the cutoff value of 56.4 % (sensitivity: 57.1 %, specificity: 90.7 %).

**Fig. 3:**
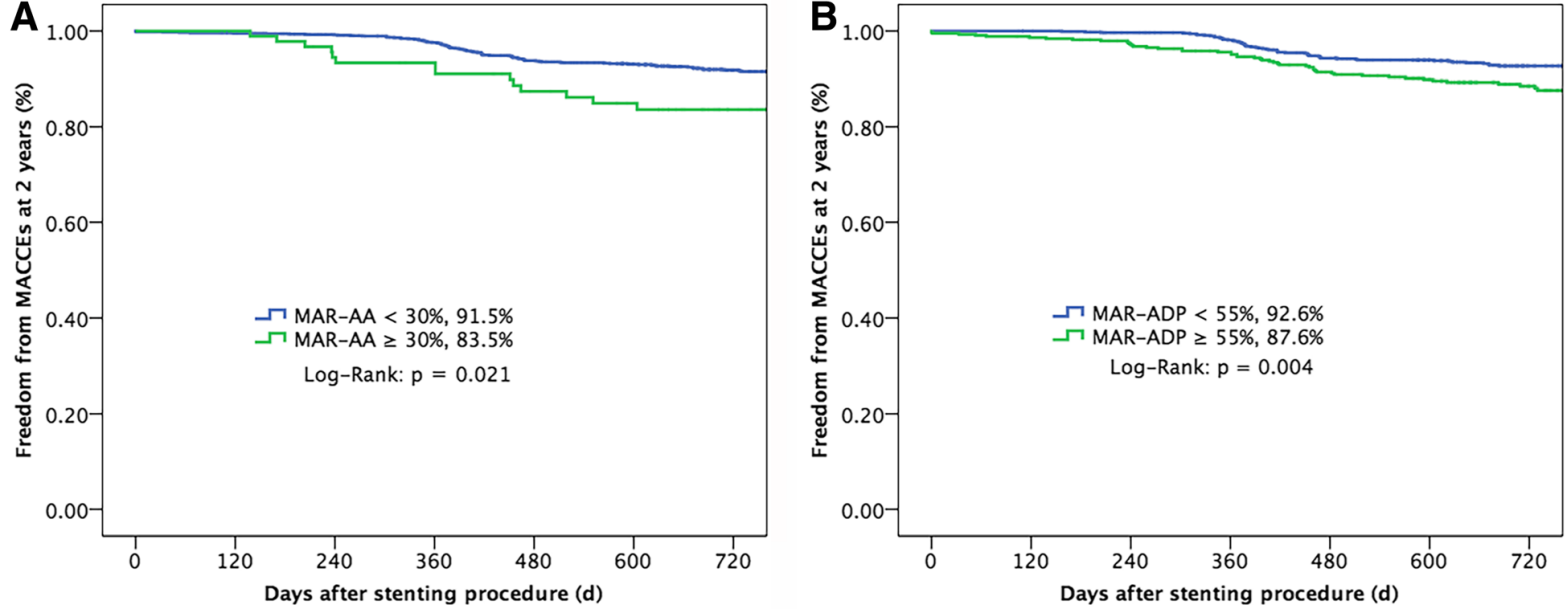
MACCE-free survival rate at 2 years. Freedom from adverse cardiovascular and cerebrovascular events at 2-year follow-up **a** between AA-MAR% ≥ 30 % (*green line*) and AA-MAR% < 30 % (*blue line*), **b** between ADP-MAR% ≥ 55 % (*green line*) and ADP-MAR% < 55 % (*blue line*). Abbreviations are showed in Fig. 1

There were 15 (16.5 %) composite MACCEs in the HPR on aspirin group and 106 (8.5 %) in the NPR on aspirin group (*p* = 0.021, Fig. 3a), mainly driven by the higher rates of all cause death (7.7 %) and MI (9.9 %) in the HPR on aspirin group compared with those in the NPR on aspirin group (1.6 %, *p* = 0.002; 1.9 %, *p* < 0.001). Similarly, the rate of MACCE was 12.4 % in the HPR on clopidogrel group, higher than 7.4 % in the NPR on clopidogrel group (*p* = 0.004, Fig. 3b), mainly due to higher rates of all cause death (5.3 %) and MI (4.8 %) in the HPR on clopidogrel group.

No differences in all bleeding and hemorrhagic stroke were observed between NPR on aspirin group and HPR on aspirin group, without significant difference found between NPR on clopidogrel group and HPR on clopidogrel group.

## Discussion

The major findings of this prospective study were that: 1) There were 6.8 % identified with high platelet reactivity on aspirin, and 32.9 % with high platelet reactivity on clopidogrel among patient undergoing PCI; 2) high platelet reactivity on both aspirin and clopidogrel were independent predictors of 2-year stent thrombosis following DESs implantation; and 3) there were no significant association between high platelet reactivity on aspirin/clopidogrel and any bleeding/ hemorrhagic stroke.

Antiplatelet therapy is the cornerstone of preventing stent thrombosis in patients after DESs implantation, but at the cost of higher bleeding risk, which has been the Achilles’ heel for these patients. Platelet function test focusing on the individual’s response to antiplatelet therapy, was expected to select the most optimal agents and dosage for these patients, in order to reduce the risk of stent thrombosis and bleeding. Indeed, the ADAPT-DES (Platelet reactivity and clinical outcomes after coronary artery implantation of drug-eluting stents) study, prospectively enrolling 8665 patients after PCI, showed that high platelet reactivity on clopidogrel was an independent predictor of stent thrombosis, but was also protective against clinically relevant bleeding. However, several other studies with different population had the conflicted results [6, 8]. The present study with a novel method confirmed the data from ADAPT-DES, indicating that platelet function test, combining with clinical and procedural factors, could be at least useful for predicting stent thrombosis for PCI patients.

In contrast with our findings, ADAPT-DES study did not identify the incremental stent thrombosis in patients with high platelet reactivity on aspirin. This difference might be due to the differences in population, platelet testing methods, as well as duration of follow-up. Indeed, there are other clinical factors to affect platelet reactivity, such as current smoking, diabetes, and hyperlipidemia, which may increase the incidence of high platelet reactivity on aspirin and the risk of adverse clinical outcomes. However, multivariable Cox proportional hazard model was used in the present study to demonstrate that high platelet reactivity on aspirin was the independent predictor of stent thrombosis, apart from clinical and procedural factors. Certainly, this new findings need be verified in the further clinical trials.

Although the preliminary association between high platelet reactivity and stent thrombosis was established, several randomized clinical trials on adjust treatment based on platelet function test in PCI patients had the negative results [16–18]. In the ARCTIC (The Assessment by a Double Randomization of a Conventional Antiplatelet Strategy versus a Monitoring-guided Strategy for Drug-Eluting Stent Implantation and of Treatment Interruption versus Continuation 1 Year after Stenting) study [16], 2440 PCI patients were randomized to platelet function monitoring group with therapy adjustment if necessary, or conventional strategy without monitoring. After 1-year follow-up, there were no significant improvements in clinical outcomes with platelet function monitoring, compared with conventional antiplatelet therapy, which was in line with other studies [17, 18]. The negative results might be due to the low-risk population, low event rate, and inclusion of periprocedural MI, which could not be prevented by platelet function test. Of note, all these studies were performed with VerifyNow, and other platelet assays such as PL-11 require further investigation. In summary, the current randomized studies do not support the routine platelet function tests to adjust treatment for PCI patients.

Just as mentioned above, there are several methods of platelet test assessing platelet reactivity. In the POPULAR (Do Platelet Function Assays Predict Clinical Outcomes in Clopidogrel-Pretreated Patients Undergoing Elective PCI) study [19], the largest head-to-head comparison among platelet test methods, only light transmittance aggregometry (LTA), VerifyNow, and Plateletworks had the modest association with the primary end point (composite of all-cause death, nonfatal acute myocardial infarction, stent thrombosis, and ischemic stroke). However, none of these tests could identify patients at the higher risk of bleeding after PCI. Moreover, the study by Lemesle G [20] showed poor agreement between LTA, VerifyNow and vasodilatator-stimulated phosphoprotein for clopidogrel low-response assessment. Therefore, platelet function assays were not equally interchangeable and the correlations among them were varied. PL-11 by “sequentially platelet counting method” gives more exact and stable results of platelet counting before and after the addition of agonists in the citrated whole blood samples, which has been demonstrated to be correlated with LTA and VerifyNow [11]. Consequently, sequentially platelet counting method by PL-11 would bean optional device for platelet function tests.

Another two questions need to be discussed further. First, Sheiban et al. [21] reported that PCI on unprotected left main (ULM) disease had a safe long-term (more than 10 years) outcome despite of using first-generation DES, with low rates of recurrent events due to index revascularization. A total of 167 patients with ULM disease were involved in our study, in which 100 patients (59.9 %) received second-generation DES implantation and 67 patients (40.1 %) with first-generation DES. There was no significant difference in MACCE between first- and second-generation DES, might due to a relative small population. Second, a recent meta-analysis [22] showed that shorter DAPT duration was associated with higher rates of MI, lower rates of major bleeding, and similar rates of stent thrombosis, and cardiovascular mortality. In our study, all patients received 100 mg/day aspirin for life and clopidogrel (75 mg/day) for at least 12 months after the intervention, without shorter DAPT duration (less than 12 months). An individualized patient approach to DAPT duration by balancing risks of bleeding and stent thrombosis was recommended. Therefore, the impact of platelet reactivity test guided DAPT duration on PCI patients will be explored in our next study.

Our data confirm the previous evidence from ADAPT-DES and add some novel findings. First, high platelet reactivity on aspirin was associated with stent thrombosis after drug-eluting stent implantation apart from clopidogrel, which was in accordance to the clinical hypothesis. Second, the associations between platelet function tests with 1-year thrombotic events were explored in most previous studies, but our study showed the reduced 2-year stent thrombosis with high platelet reactivity for the first time. Finally, it is the first time for PL-11 used in clinical trials, and PL-11 might bean optional device for platelet function tests.

### Study limitation

The current study has several limitations. First, none of the 1331 patients had received therapy adjustment according to platelet function tests, and it was not available for evaluating the effects of platelet function tests-guided therapy adjustment on cardiovascular outcome. Second, all enrolled patients underwent platelet function tests before DES implantation, and it might not be possible to conduct a repeat platelet test in consideration of the short hospitalization period in patients after PCI. Third, there was the lack of platelet function tests post-discharge, and we could not find time-dependent changes in platelet activity.

## Conclusions

This prospective study showed high platelet reactivity on both aspirin and clopidogrel were associated with incremental stent thrombosis following drug-eluting stent implantation.

## Additional file

Additional file 1: Figure S1. Receiver-operating characteristic curve predicting stent thrombosis. (TIFF 5134 kb)

## Abbreviations

AA: Arachidonic acid
ACS: Acute coronary syndromes
ADP: Adenosine diphosphate
ARC: Academic Research Consortium
BARC: Bleeding Academic Research Consortium
CK: Creatine kinase
DESs: Drug eluting stents
HPR: High platelet reactivity
LTA: Light transmittance aggregometry
MACCE: Major adverse cardiovascular and cerebrovascular events
MAR: Maximal aggregation ratio
MI: Myocardial infarction
NPR: Normal platelet reactivity
PCI: Percutaneous coronary intervention
ROC: Receiver-operating characteristic
TVR: Target vessel revascularization
