# Supplementary figures and images for: High platelet reactivity affects the clinical outcomes of patients undergoing percutaneous coronary intervention
Source: BMC Cardiovasc Disord. 2016 Nov 29;16:240. doi: 10.1186/s12872-016-0394-0 (PMC5126985; doi:10.1186/s12872-016-0394-0)

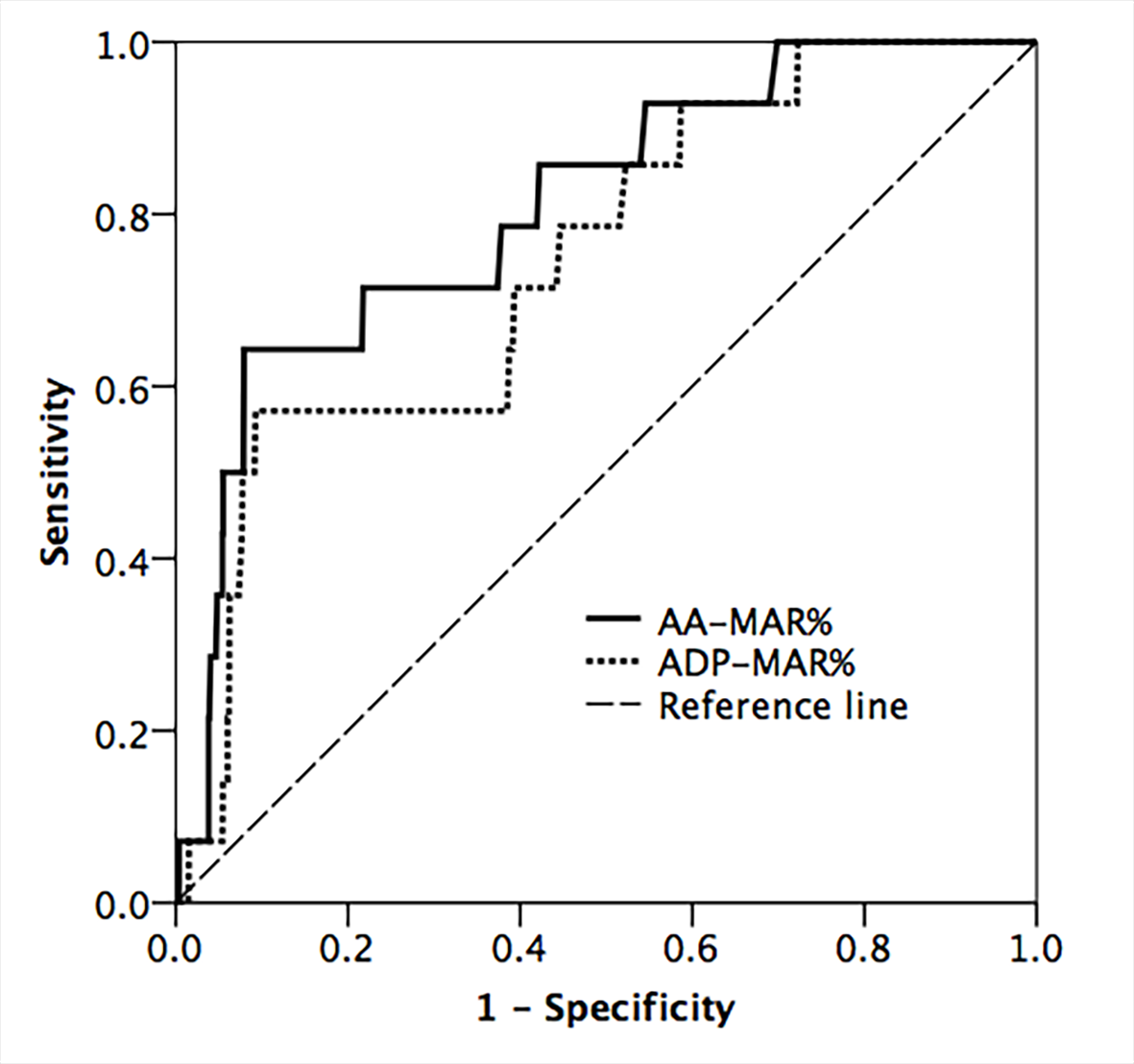

Supplement: Additional file 1: Figure S1. — Receiver-operating characteristic curve predicting stent thrombosis. (TIFF 5134 kb) [file 12872_2016_394_MOESM1_ESM.tiff]
